# Role of the prefrontal cortex in prosocial and self-maximization motivations: an rTMS study

**DOI:** 10.1038/s41598-021-01588-6

**Published:** 2021-11-16

**Authors:** Oksana Zinchenko, Olga Savelo, Vasily Klucharev

**Affiliations:** 1 Institute of Cognitive Neuroscience, Centre for Cognition and Decision Making, National Research University Higher School of Economics, Krivokolenniy Sidewalk, 3a, Moscow, Russian Federation 101000; 2 Department of Psychology, National Research University Higher School of Economics, Armyansky Sidewalk, 4, Moscow, Russian Federation 101000; 3 International Laboratory for Social Neurobiology, Institute of Cognitive Neuroscience, National Research University Higher School of Economics, Krivokolenniy Sidewalk, 3a, Moscow, Russian Federation 101000

**Keywords:** Social neuroscience, Neuroscience, Cognitive neuroscience, Psychology, Human behaviour

## Abstract

More than a decade of neuroimaging and brain stimulation studies point to a crucial role for the right dorsolateral prefrontal cortex (rDLPFC) in prosocial behavior. The intuitive prosociality model postulates that the rDLPFC controls intuitive prosocial behavior, whereas the reflective model assumes that the rDLPFC controls selfish impulses during prosocial behavior. The intuitive prosociality model implies that the transient disruption of the rDLPFC should increase voluntary transfers in both dictator and generosity games. In contrast, the reflective model suggests that the transient disruption of the rDLPFC should decrease transfers in the dictator game, without affecting voluntary transfers in the generosity game, in which selfish motives are minimized. The aim of this paper was to compare predictions of the intuitive and reflective models using the classic dictator game and generosity game and continuous theta burst stimulation (cTBS). In this study, two groups of healthy participants (dictators) received either cTBS over the rDLPFC or right extrastriate visual areas. As shown by the results, the transient disruption of the rDLPFC significantly promoted prosocial motives in the dictator game only, particularly in the trials with the lowest dictator’s costs. These findings partially support the notion that the rDLPFC controls intuitive prosocial behavior.

## Introduction

Standard economic models have traditionally assumed that economic decisions are motivated solely by “utilitarian utility” and are not directly influenced by prosocial motivations^1^. However, many behavioral and neuroimaging studies have suggested that prosocial norms play an essential role in economic decision-making^2,3^. Much of this evidence comes from studies using behavioral economics games, such as the dictator game^4^, in which player 1 (the dictator) can offer any fraction of an endowment to player 2 (receiver), keeping the rest for him- or herself. In this game, a rational and selfish player 1 would distribute no money to player 2. However, in most studies, the majority of players contribute some money^5^, which indicates an important role for altruism and inequity aversion in economic decision-making^6^. The current study aimed to further investigate the causal mechanisms underlying the neural bases of prosocial behavior and to further test the prediction of the key theoretical models in the field.

Previous neuroimaging studies have suggested that dorsolateral prefrontal cortex (DLPFC) activity during the dictator game, as well as in other games, is involved in choices between selfish and prosocial options^7–13^. In previous research, continuous theta burst stimulation (cTBS) of the right DLPFC (rDLPFC) via inhibitory repetitive transcranial magnetic stimulation (rTMS) led to more generous behavior in the dictator game^14^. Similarly, cathodal transcranial direct current stimulation (tDCS) increased voluntary transfers in the dictator game relative to a sham condition, whereas anodal tDCS decreased voluntary transfers in the game^12^. The cortical thickness of the DLPFC was found to be negatively correlated with the level of giving in the dictator game^15^. Thus, there is evidence that the DLPFC reduces, rather than promotes, prosocial behavior in the dictator game, with people reducing prosocial behavior during social exchanges when this natural predisposition is overridden for various strategic reasons to preserve their self-interests^16,17^. As summarized by Yamagishi et al.^15^, the “intuitive prosociality model” suggests that the DLPFC controls intuitive prosocial behavior. Such a view of prosocial behavior implies that prosociality is an intuitive mechanism of human social life that reflects the evolutionary adaptation of reward processing in cooperative social contexts^16,17^. Indeed, many behavioral studies strongly suggest that prosocial behavior arises from intuitive preferences (for a review, see^18^).

On the other hand, some studies have demonstrated that inhibitory tDCS and rTMS of the rDLPFC lead to stronger maximization of the budget in the ultimatum game, where the receiver can accept or reject the offer of the dictator^8,9,12,19,20^. Thus, the alternative “reflective model” suggests that the DLPFC may control selfish impulses during prosocial behavior^15^. For example, in the ultimatum game, conflicts between social and self-interest motivations are exhibited in higher rDLPFC activity^21^. Prosociality models suggest that before acting prosocially, individuals must overcome selfish impulses^16,22,23^. In prosociality models, selfishness constitutes an intuitive, emotional tendency, and reflective control is required to overcome this tendency and demonstrate prosociality (for details, see^18^).

The current study addressed inconsistencies in the literature regarding rDLPFC involvement in the control of particular motivations during decision-making. Specifically, we investigated whether the rDLPFC is involved in self-maximization (according to the intuitive prosociality model) or in the implementation of prosocial norms (according to the reflective model).

The choices made by players in the dictator game are considered a reflection of social preferences, unaffected by strategic considerations^11,22,24,25^. However, the dictator game does not control for the motivation to maximize the dictator’s own budget. To control this motivation, in the current study, we used a modified generosity game^26^. In the generosity game, the dictator chose the size of the pie (i.e., the monetary amount to be divided between two players), knowing that his/her own share was fixed. Hence, in the generosity game, the dictator was able to give more money to another player without incurring much monetary cost. Thus, this modified generosity game reduced the self-maximization motivation.

It should be noted that the generosity game does not fully eliminate self-maximization motivation. For example, the theory of inequality aversion^25^ implies that a greater difference between dictators’ and recipients’ payoffs should discount dictators’ payoffs, even though their share is fixed. Thus, the generosity game may trigger conflicts between “generous” prosocial motivations and equity-seeking behavior. Therefore, to control for different motivations, additional experimental conditions, including a range of different possible pie sizes, are often included in the generosity game. Nevertheless, during the generosity game, most dictators maximize recipients’ payoffs, with a substantial number of participants preferring equal shares and only a minority minimizing recipients’ payoffs^26^. Overall, the generosity game significantly reduces the trade-off between self-interest and prosocial motivation as compared to the dictator game. Indeed, in a previous study, transfers to a recipient in the generosity game were larger than those in the dictator game^27^.

Based on the intuitive prosociality model, we hypothesized that inhibitory cTBS of the rDLPFC would stimulate prosocial motives and increase voluntary transfers in both the (a) dictator and (b) generosity games (Hypothesis I). Alternatively, based on the reflective model, we hypothesized that inhibitory cTBS of the rDLPFC would stimulate selfish behavior and, consequently, (a) decrease voluntary transfers in the dictator game while (b) not affecting voluntary transfers in the generosity game in which the dictator’s budget was fixed (Hypothesis II).

## Results

**Figure 1:**
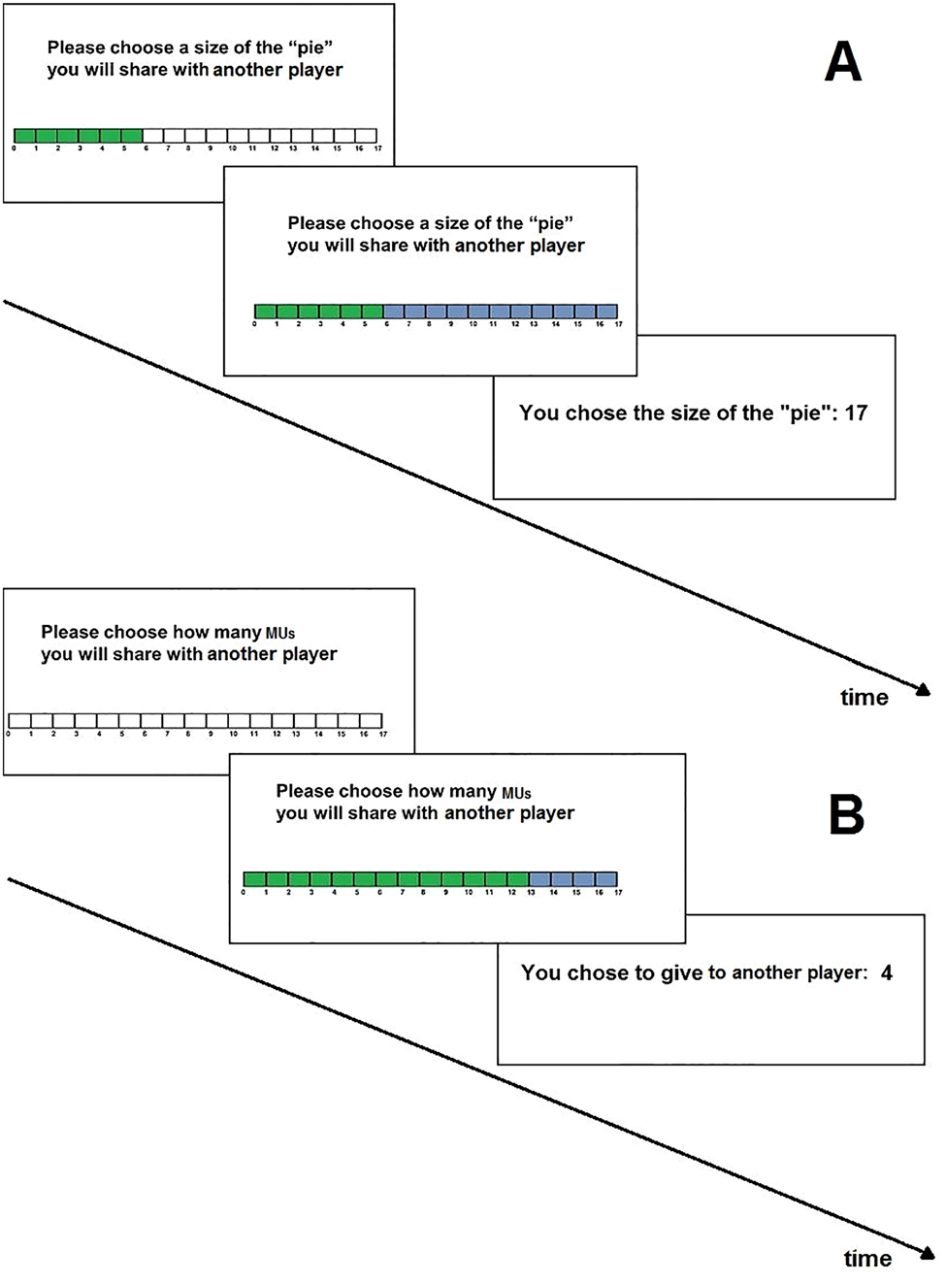
The timelines of the sample trials in (**A**) the generosity game and (**B**) dictator game. Blue indicates the size of the pie (generosity game) or the number of MUs shared with player 2 (dictator game). Green indicates player 1’s fixed share of the pie (generosity game) or the number of MUs kept by player 1 (dictator game).

To test our hypotheses, we compared the effect of offers made during the dictator and generosity games on cTBS of the rDLPFC and the right extrastriate visual (rMT/V5) areas among two groups of participants. After either rDLPFC or rMT/V5 stimulation, each subject took part in one session of the dictator game and one session of the generosity game. In the generosity game, the participant (player 1) selected the size of a pie to be shared with player 2. Similar to the original design of the generosity game^19^, the identities of the players remained unknown, with no personal data or photographs of the players presented during either the generosity or dictator game. The participants played against remote players (player 2: *N* = 349; first-, second-, and third-year undergraduate students) who had been contacted prior to the study and assigned a unique sequential identification number. In the generosity game, the payoff for player 1 was constant (x = 6 monetary units [MUs]). To control for different motivations, we used three treatments: (1) trials favoring player 1 (FP1 trials), in which the dictator was permitted to choose a pie size of between 7 and 11 MUs; (2) trials favoring player 2 (FP2 trials), in which the dictator can choose a pie size of between 13 and 17 MUs; and (3) trials with an equal-split treatment (E trials), in which the dictator can choose a pie size of between 7 and 17 MUs (Fig. 1A and Methods for details).

The dictator game consisted of 11 trial types (endowment = 7–17 MUs). In the dictator game, the participants (player 1) could select any portion of the endowment to share with player 2 (Fig. 1B and Methods for details). The payoff structure of the generosity and dictator games allowed prosocial behavior in the two games to be compared. The FP1 trials in the dictator game, with a total endowment of 7–11 MUs, were similar to the FP1 trials in the generosity game. The FP2 trials in the dictator game, with a total endowment of 13–17 MUs, were similar to the FP2 trials in the generosity game. All E trials in the dictator game were similar to the E trials in the generosity game. We analyzed the allocations to the recipients in the dictator game and the offered pie size (in MUs) in the generosity game, which were measured using the same scales and hence required no normalization.

### Overall effects of cTBS on the allocations to receivers in the dictator and generosity games

A Kolmogorov–Smirnov test showed that the offers in both stimulation groups were not normally distributed: rDLPFC group, *p* = 2.9 × 10^−223^; MT/V5 group, *p* = 1.2 × 10^−214^). Therefore, we used ordinal regression for parameter estimation and a nonparametric Mann–Whitney *U*-test for a post-hoc analysis. The ordinal regression model (Model 1) included the factors *Game* (generosity game/dictator game) and *Stimulation* (cTBS of the rDLPFC/cTBS of the MT/V5) as categorical variables and the allocation to receivers and offered pie size (in MUs) as the dependent variable. The chi-square statistic for the model fit was equal to 1813.50 (*p* < 0.001), suggesting that the final model was significantly improved compared to the baseline intercept-only model. The model explained around 11% of the variance (Cox–Snell’s *R* = 0.11). The ordinal regression model reveled significant main effects of the factors *Game* (odds ratio of 4.12; 95% confidence interval [CI]: 3.74 to 4.54) and *Stimulation* (odds ratio of 0.87; 95% CI: 0.82 to 0.93) on the allocations to player 2 and a significant *Game* × *Stimulation* interaction (odds ratio of 1.34; 95% CI: 1.17 to 1.53; (see details in Table 1; for descriptive statistics see supplementary Table S1).

**Table 1:** Ordinal regression analysis (Model 1), showing the effect of stimulation in the FP1/FP2 trials in the dictator and generosity games.

	Estimate	Wald	Odds ratio	LL	UL	*p* value
Game	1.4163	815.5072	4.12	3.74	4.54	< 0.001
Stimulation	− 0.1398	19.4715	0.87	0.82	0.93	< 0.001
Game × Stimulation	0.2934	18.3072	1.34	1.17	1.53	< 0.001

Here and below, *LL* lower confidence limit, *UL* upper confidence limit.

Thus, the ordinal regression analysis confirmed that after cTBS perturbation of the rDLPFC, allocations to player 2 significantly increased when compared with perturbation of the MT/V5, with a mean allocation to player 2 after rDLPFC perturbation of 5.49 MUs and a mean allocation to player 2 after MT/V5 perturbation of 5.26 MUs. The significant effect of the factor *Game* indicated that, as expected, during the generosity game, allocations to player 2 were larger than during the dictator game. The mean average allocation in the generosity game was 7.43 MUs, whereas it was 4.81 MUs in the dictator game. The significant positive *Game* × *Stimulation* interaction pointed to a differential effect of cTBS on allocations in the dictator and generosity games.

**Figure 2:**
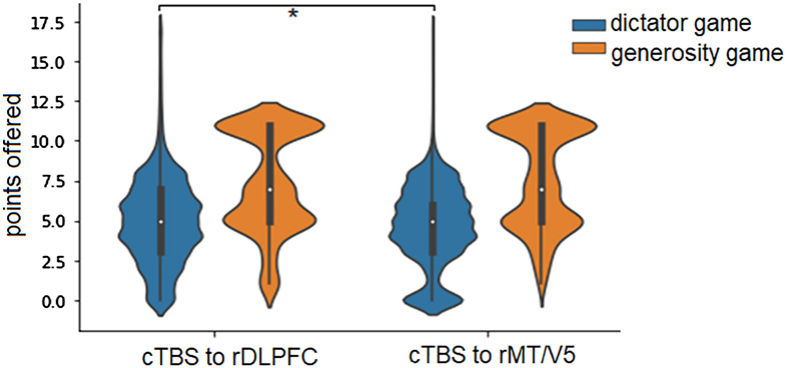
Allocations to recipients in the dictator game and the offered pie size in the generosity game. The blue violin plot indicates the results of the dictator game, and the orange violin plot indicates the results of the generosity game. The data correspond to cTBS under the following experimental conditions: rDLPFC = cTBS of the rDLPFC; MT/V5 = control cTBS of the MT/V5. *Note*: The bottom of the violin plot indicates the minimum, the lower bar of the boxplot denotes the first quartile (Q1), the white dot denotes the median, the higher bar of the boxplot denotes the third quartile (Q3), and the top of the violin plot indicates the maximum. An asterisk (*) indicates significance at *p* < 0.05 (Mann–Whitney U test).

To further explore the nature of the *Game* × *Stimulation* interaction, we performed pairwise comparisons using a Mann–Whitney Test. As shown by the results, in the dictator game, cTBS of the rDLPFC significantly increased allocations compared to rMT/V5 stimulation: *M*_rDLPFC_ = 4.99 ± 2.77, *M*_MT/V5_ = 4.63 ± 2.58; *U* = 17,554,854, *Z* =  − 4.589, *p* < 0.0001. No significant differences in the allocations between the rDLPFC and rMT/V5 stimulation groups were observed in the generosity game: *M*_rDLPFC_ = 7.31 ± 3.17; *M*_rMT/V5_ = 7.55 ± 3.09; *U* = 1,345,339.5; *Z* =  − 0.97; *p* = 0.33. Figure 2 illustrates the distributions of the data points in the rDLPFC and rMT/V5 stimulation groups in the generosity and dictator games. As can be seen, the offers after cTBS of the rMT were more skewed toward zero offers than after cTBS of the rDLPFC.

### The role of inequality aversion in the behavioral effects of cTBS

**Figure 3:**
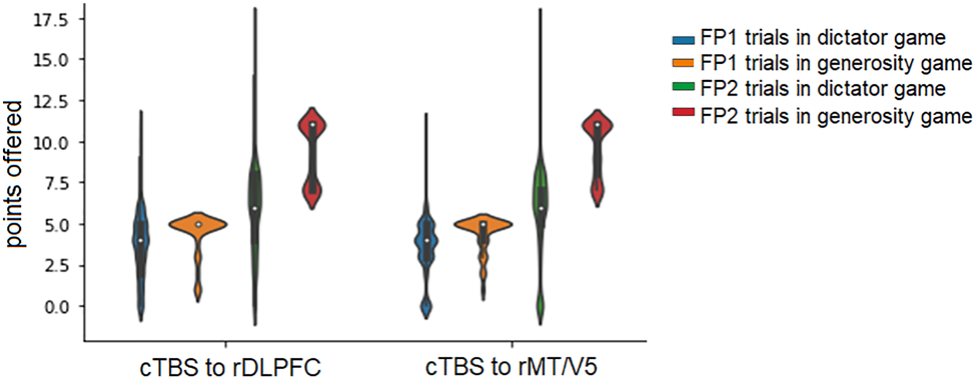
Allocations to recipients in the dictator game and offered pie size in the generosity game in the FP1 and FP2 trials. The blue violin plot indicates the data points corresponding to the FP1 trials in the dictator game, the orange violin plot corresponds to the FP1 trials in the generosity game, the green violin plot corresponds to the FP2 trials in the dictator game, and the red violin plot corresponds to the FP2 trials in the generosity game. The data corresponding to cTBS of the rDLPFC are depicted on the left, and the data corresponding to cTBS of the MT/V5 are depicted on the right. *Note*: The top and bottom of the violin plot indicate the maximum and minimum, respectively, the lower bar of the boxplot denotes the first quartile (Q1), the white dot denotes the median, and the higher bar of the boxplot denotes the third quartile (Q3).

The results of the ordinal regression analysis of Model 1 suggested that cTBS of the rDLPFC significantly increased allocations in the dictator game compared to the rMT/V5 stimulation, whereas no such trend was observed in the generosity game. Therefore, we next checked whether the behavioral effects of cTBS were modulated by inequality aversion. We focused on the effect of cTBS in the FP2 trials, in which the dictators could face “disadvantageous” inequality, and the FP1 trials, in which the dictators could face “advantageous” inequality. To construct the additional ordinal regression model, we recoded the data to create the following dummy variables: allocations to recipients in the FP1 trials in the dictator game (*FP1 in DG*), allocations to recipients in the FP2 trials in the dictator game (*FP2 in DG*), allocations to recipients in the FP1 trials in the generosity game (*FP1 in GG*), and allocations to recipients in FP2 trials in the generosity game (*FP2 in GG*)*.* We then used an ordinal regression model (Model 2), the polytomous universal model (PLUM), with the following factors as categorical variables: *Stimulation* (cTBS of the rDLPFC/cTBS of the MT/V5) and *Trial Type* (*FP1 in DG*/*FP2 in DG*/*FP1 in GG*/*FP2 in GG*). In Model 2, the chi-squared statistic for the PLUM ordinal regression model fit (6446.63, *p* < 0.001) suggested that the final model was significantly improved as compared to the baseline intercept-only model. The model explained around 39% of the data (Cox–Snell’s *R* = 0.39). The results of the ordinal regression model are presented in Table 2 and show that the main effects (*Stimulation* and *Trial Type*) were significant. Notably, after Bonferroni correction (0.05 ÷ 4), the interaction in the *Stimulation* × *FP1 trials* remained significant in the dictator game, whereas the interaction in the *Stimulation* × *FP2 trials* just approached significance. Figure 3 shows that after cTBS of the rDLPFC, the offers in the TP1 and TP2 trials were more skewed toward maximum possible offers than after cTBS of the rMT. This confirmed that the allocations to player 2 were significantly larger after cTBS of the rDLPFC than after cTBS of the MT/V5, particularly in the FP1 trials in the dictator game (for descriptive statistics see supplementary Table S2). Therefore, we observed the strongest effects of cTBS in the trials in the dictator game with the lowest endowment and, therefore, the lowest dictator’s costs.

**Table 2:** Ordinal regression analysis (Model 2), showing the effect of stimulation in the FP1/FP2 trials in the dictator and generosity games.

	Estimate	Wald	Odds ratio	LL	UL	*p* value	Bonferroni-corrected *p* value
Stimulation	− 0.295	7.507	0.745	0.603	0.920	0.006	
FP1 in DG	− 5.181	3016.594	0.006	0.005	0.007	< 0.001	
FP1 in GG	− 4.462	1514.658	0.000	0.009	0.014	< 0.001	
FP2 in DG	− 2.921	1048.599	0.054	0.045	0.064	< 0.001	
FP2 in GG^a^	0		1			N/A	
Stimulation × FP1 in DG	0.518	19.468	1.679	1.334	2.113	< 0.001**	0.00004
Stimulation × FP1 in GG	0.364	5.834	1.439	1.071	1.934	0.016	0.064
Stimulation × FP2 in DG	0.295	6.300	1.343	1.067	1.690	0.012	0.048
Stimulation × FP2 in GG^a^	0		1			N/A	

^a^Represents reference category; DG—dictator game; GG—generosity game; FP1—FP1 trials; FP2—FP2 trials.

**p = 0.00001.

## Discussion

We observed a significant effect of cTBS of the rDLPFC on the behavior of player 1 in the dictator game, which is partially consistent with Hypothesis I. According to the intuitive prosociality model, the rDLPFC serves as a hub for strategic reasoning and, if left alone, controls the intuitive drive for prosociality. According to our findings (Model 1), offline cTBS of the rDLPFC increased allocations to recipients in the dictator game compared to the control condition. These results partially support the intuitive prosociality model and imply that inhibitory cTBS of the rDLPFC should stimulate prosocial motivation and increase voluntary transfers in both dictator and generosity games. A more detailed analysis (Model 2) revealed a significant behavioral effect of brain stimulation in the dictator game in the trials with relatively small endowments (i.e., 7–11 MUs) and a similar, although not significant, trend in comparable trials in the generosity game, in which the participants increased their allocations to player 2 after perturbation of the rDLPFC as compared to perturbation of the rMT/V5 area. Thus, our results only partly support the intuitive prosociality model (Hypothesis I), implying that inhibitory cTBS of the rDLPFC should stimulate prosocial motivation and increase voluntary transfers in economic games.

Our results are in line with those of a previous study, which showed that suppression of the rDLPFC increased generosity in the dictator game^14^. Christov-Moore et al.^14^ showed that cTBS of the rDLPFC increased the mean offer to players with high, but not low, socioeconomic status. In another social context in which the dictator game followed the ultimatum game, perturbation of the rDLPFC reduced fair behavior toward opponents^13^. A functional magnetic resonance imaging (fMRI) study showed that when the dictator game followed the ultimatum game, activation of the rDLPFC may be involved in inhibiting emotional responses to unfairness and reducing retaliation against unfair behavior^28^. Together, these results suggest that the role of the rDLPFC in the control of prosocial motivation depends upon the social context.

In the current study, we observed a strong effect of rDLPFC stimulation, particularly in the trials in the dictator game with the smallest endowments. Two previous comprehensive meta-analyses suggested that lower stakes consistently increase donations in the dictator game as compared to higher stakes^5,29^. Theoretically, smaller endowments decrease the monetary value (i.e., the amount of MUs) of an offer as compared to higher endowments, for example, offering 4 MUs of an 8 MU endowment (50%) compared to offering 8 MUs of a 16 MU endowment. This lower cost of giving may enhance the willingness of the dictator (player 1) to be more generous when splitting endowments than in higher endowment conditions in which the cost of giving is much higher^29–32^. Furthermore, the higher cost of giving (larger endowment size) may make monetary concerns more salient than normative concerns and influence the offer size at a lower cost of giving^33^. Given the aforementioned findings, in the current study, the intrinsic motivation to generously split the endowments should have been highest in the trials in the dictator game with the smallest endowments. Furthermore, perturbation of the rDLPFC should have encouraged prosocial behavior and increased voluntary transfers, particularly in the trials in the dictator game with the smallest costs of giving.

It has been widely assumed in neuroscience that decisions are based on decision values assigned to options, such that neural decision-making mechanisms compare these values when making a choice^34–36^. Many studies have linked activity in the DLPFC with modulation and computation of decision values^37–40^. Particularly, if self-control is needed, neural decision value signals seem to be modulated by the DLPFC, which perhaps does not encode these values per se^20,41–43^. Our results point to the possibility that inhibitory cTBS of the rDLPFC promotes prosocial motives by liberating high decision values assigned to generous voluntary transfers in the dictator game, especially in trials with lower dictator’s costs. Recent studies also suggested that the DLPFC is involved in value computation and learning^44,45^. For example, Tsutsui et al.^44^ demonstrated that the DLPFC is involved in flexible object-specific valuations derived from recent experience. In a two-armed bandit task, Morris et al.^45^ showed that the DLPFC encodes the probability that one action value is better than an alternative action. In our study, cTBS of the DLPFC may have modulated (action) values or the values of prosocial decisions. Unfortunately, standard dictator and generosity games are not well suited to model dynamic changes in action values. If cTBS reduced the values of the MUs (or modulated action values) in the present study, we should have seen cTBS increase prosocial decisions in all the trial types in both the dictator and generosity games. Instead, cTBS of the rDLPFC significantly promoted prosocial motives in the dictator game only, particularly in the trials with the lowest dictator costs. Clearly, further studies and new behavioral paradigms are needed to test an alternative interpretation of our results.

Surprisingly, we observed only a small and insignificant trend of cTBS effects in the generosity game, in which the proposer’s (player 1) own share was fixed. Our results indicate that cTBS of the rDLPFC may stimulate prosocial motives in the dictator game, but mainly in the presence of stronger conflict between prosocial and selfish motives during the game. Güth et al.^26^ suggested that different motivations may drive decision-making in the generosity game. In their study, each proposer (player 1) faced a conflict between being generous, efficiency seeking and equity seeking. In two treatments (FP1 trials and FP2 trials, called Fx trials and Fy trials in Güth et al.’s study^26^), selecting the “generous” choice maximized the absolute difference between the players’ payoffs. Thus, generosity and inequality aversion might lead to different decisions in the generosity game, with FP1 trials potentially leading to advantageous inequity, as player 1 receives more than player 2, and FP2 trials possibly increasing disadvantageous inequity aversion, as player 1 receives less than player 2^25,46,47^. Indeed, a previous study showed that when there was no conflict between generosity and equity seeking (E trials) in the generosity game, proposers tended to most frequently choose 12 or 17 MU pie sizes, suggesting that some people act as if they are inequity averse^26^. Interestingly, we found a stronger effect of cTBS of the rDLPFC on the allocations to player 2 in the FP1 trials in the dictator game. This suggests that the effect of cTBS stimulation was particularly pronounced in the trials in the dictator game with the lowest possible “disadvantageous” inequity. This result points to the possibility that perturbation of the rDLPFC does not simply induce inequity aversion motivation (or “disadvantageous” inequity aversion^48^). If this were the case, as compared to the control condition, cTBS of the rDLPFC would be expected to have had clear behavioral effects in both the FP1 and FP2 trials in both the generosity and dictator games. However, we found a near-significant effect of cTBS in the FP2 trials in the dictator game but not in the generosity game.

Further studies with a larger sample size and different subgroups of participants may help to disentangle the differential effects of cTBS of the rDLPFC on different prosocial motivations. Follow-up studies with a large number of trials are also clearly needed to explore possible differential effects of TMS perturbation on the rDLPFC in different trial types in the generosity game. As previous studies demonstrated that the effects of DLPFC stimulation are highly context dependent^12,14^, what should be investigated in future studies.

This is the first brain stimulation study to focus on the generosity game. Using the dictator game, Christov-Moore et al.^14^ demonstrated that TMS of the rDLPFC increased prosociality toward players with a high socioeconomic status. However, in another social context, when the dictator game followed the ultimatum game, perturbation of the rDLPFC reduced fair behavior toward opponents^13^. In an fMRI study where the dictator game followed the ultimatum game, the authors concluded that activation of the rDLPFC may be involved in inhibiting emotional responses to unfairness and reducing retaliation against unfair behavior^28^. Previous studies were unable to test fully whether brain stimulation of the DLPFC leads to disinhibition of prosocial impulses, as suggested by Christov-Moore and Iacoboni^49,50^. We showed that significant disinhibition of prosocial impulses takes place in the dictator game only if there is a conflict between self-maximization and prosocial motives and that the same does not occur in the generosity game where these conflicts are lower. To shed light on the role of the self-maximization motive in prosocial decisions, we compared the behavior of the same participants in the dictator game and generosity game. This type of approach may provide a novel tool to study prosocial impulses in the dictator game using the generosity game as a control condition.

Similar to many previous studies^11,15,51,52^, in our study, the participants played the dictator and generosity games using a simple graphical interface, with no photographs of their counterparts (i.e., player 2). In contrast, in some brain stimulation research, player 1 was shown a photograph of player 2 in the dictator game^14^. Previous TMS studies on prosocial behavior in the dictator game used different target coordinates. Christov-Moore et al.^14^ utilized the results of a previous fMRI study (MNI coordinates 40, 24, and 38) in a cTBS study, and Ruff et al.^12^ used coordinates from another fMRI study (MNI coordinates 28, 26, 24/38, 38, and 28) for tDCS stimulation. While, Müller-Leinß et al.^13^ applied TMS to the middle part of the middle frontal gyrus using an anatomically based approach. As previous studies investigated different subregions of the DLPFC, future studies should investigate further a possible regional specialization within the DLPFC.

Future studies could also use a modified generosity game to study the role of social comparison. For instance, the socioeconomic status of participants could be explicitly stated during the generosity game. For example, based on previous findings, we would expect a significant effect of rDLPFC stimulation during a modified generosity game (as compared to MT/V5 stimulation) when player 2 has a high socioeconomic status. It is also possible that fixed shares anchor players’ allocations in the generosity game. Thus, future studies could manipulate the size of the dictator’s payoff to account for the anchoring effect. Despite these limitations, our results provide new evidence for the prominent role of the DLPFC in prosocial behavior.

Overall, the present study confirms the fundamental role of the DLPFC in controlling prosocial behavior during social decision-making^8,14,47^. We showed that perturbation of the rDLPFC promotes prosocial motives in the dictator game, particularly in trials with lower dictator costs. Our results provide some support for the intuitive prosociality model (Hypothesis I), which suggests that the rDLPFC controls intuitive prosocial behavior. Conversely, our results may contradict the reflective model (Hypothesis II), which suggests that the rDLPFC exerts an inhibitory influence on self-maximization^7,8^. Further studies are clearly needed to determine the role of the rDLPFC in prosocial behavior, particularly in the generosity game. Overall, our findings represent a step forward toward greater understanding of the tendency to behave prosocially and deviate from rational self-interest.

## Methods

### Subjects

The effect size was estimated based on a recent study by Christov-Moore et al.^14^ that examined the effect of offline cTBS of the DLPFC on offers during the dictator game. We calculated the effect size for *F*-tests (analysis of variance: fixed effects, special, main effects, and interactions), with 1 − β = 0.95 and α = 0.05. In the study by Christov-Moore et al.^14^, there were 19–20 subjects in each of three groups (df = 2.107), which resulted in an effect size of *d* = 1.06. We used a between-subjects design in the TMS study. A total sample size of 40 was calculated, with 20 participants in the experimental group (rDLPFC inhibition) and 20 in a control group (MT/V5 stimulation). If the participants (players 1) shared no MUs with player 2 during at least one session, they were considered to be outliers and excluded from the analysis. The expected dropout rate was 25%. Thus, the required sample size in each group was 20 × 1.25 = 25 (females, *n* = 13) participants per group, giving 50 participants in total.

Fifty individuals aged between 18 and 27 years were invited to take part in the study (rDLPFC and rMT/V5 stimulation). Four individuals stated that they experienced highly uncomfortable sensations from the single pulse stimulations and were therefore excluded from the final sample, leaving 46 participants in total: 23 participants (females, *n* = 12; mean age = 21.82 years; SD = 1.89) in the rDLPFC group and 23 participants (females, *n* = 13; mean age = 21.61 years; SD = 2.19) in the rMT/V5 group.

All the participants were right-handed, with normal or corrected-to-normal vision and no history of neurological or psychiatric disorders. Standard exclusion criteria for TMS were used: pregnancy, metallic implants, cardiac or neurological health conditions, and specific medications.

All the participants provided written informed consent before the experiment. The TMS protocol was approved by the local ethics committee: the HSE Committee on Interuniversity Surveys and Ethical Assessment of Empirical Research.

The introduction, hypotheses, methods, and analysis plan were reviewed before the research was conducted. All methods were carried out in accordance with relevant guidelines and regulations. The materials are available on the Open Science Framework website (https://osf.io/twd7f/), and the dataset and script are available at Harvard Dataverse (https://dataverse.harvard.edu/dataverse/tmsstudy2020).

### Experimental procedures

Each subject was assigned to either the rDLPFC or rMT/V5 stimulation group and took part in one session of the dictator game and generosity game. As we used a between-subject design, each subject was assigned only to one stimulation group. The trials in both games were intermixed and randomized across subjects.

### Generosity game

In the generosity game, player 1 selected a pie size (i.e., the size of the endowment (p) that he/she was willing to share with player 2^26^. To ensure no conflict between the payoffs of either player, the payoff for player 1 was independent of the size of the pie size (p) and equal to a constant x = 6 MUs. In our study, the outcome for player 2 was equal to p = 6.

Güth et al.^26^ suggested that different motivations may drive decisions in the generosity game, with conflicts between “generous” prosocial motivations and equity-seeking behavior. To control for these, they proposed the use of three treatments, in which the range of possible pie sizes was modified in a systematic way. In the present study, the participants took part in three types of trials (treatments), with a similar task structure to that described previously by Güth et al.^26^. The compositions of the trials are described below:

1. Trial type 1 favored player 1 (FP1 trials). In this trial, the dictator was allowed to chose a pie size (the sum of which was split between the players) of between 7 and 11 MUs. In this trial, the preferences for generosity and equity were aligned. Therefore, both motivations favored an optimal size of an endowment, which was p = 11.
2. Trial type 2 favored player 2 (FP2 trials). In this trial, the dictator was allowed to chose a pie size of between 13 and 17 MUs. In this trial, equity seeking would favor p = 13, whereas generosity would favor p = 17, implying a conflict between the two motivations.
3. Trial type 3 featured an equal split treatment (E trials) design. In this trial, the dictator was allowed to chose a pie size of between 7 and 17 MUs. The only treatment that allowed the dictator to choose an equal split was p = 12.

Figure 1A illustrates a sample trial (FP2 trial) in the generosity game, in which the dictator selected a pie size of between 7 and 17 MUs.

### Dictator game

The dictator game consisted of 11 trial types, with the following sizes of endowment: 17, 16, 15, 14, 13, 12, 11, 10, 9, 8, and 7 MUs. In the dictator game, player 1 (dictator) selected the portion (y) of the endowment (p) that he/she would share with player 2. Thus, this decision influenced the dictator’s own budget, which was equal to x = p − y. Figure 1B illustrates a trial in which player 1 (dictator) shared 4 MUs with player 2. The participants were given an unlimited time in which to make their decisions. After deciding, the participant pressed the button to proceed to the next trial.

MUs were scored as points, with a conversion rate of 1 point = 20 Russian rubles (approximately equal to 0.29 U.S. dollars). The offer size in the dictator game was defined as the number of points that player 1 decided to share with player 2. In both the dictator and generosity games, each trial type (treatment) was presented 24 times, similar to Christov-Moore et al.’s study^14^. Overall, the payoff structure in the generosity and dictator games mirrored each other to allow a more detailed comparison of the behavior in the two games. The compositions of the trials were as follows:

1. Trials in the dictator game (FP1 trials), where the total endowment was equal to 7–11 MUs, were similar to the payoffs in the FP1 trials in the generosity game;
2. Trials in the dictator game (FP2 trials), where the total endowment was 13–17 MUs, were similar to the payoffs in the FP2 trials in the generosity game;
3. The payoffs in the E trials in the dictator game (E trials) were similar to the payoffs in the E trials in the generosity game.

Similar to the original design of the generosity game^26^, the identities of the players remained unknown, with neither personal data nor photographs of the players presented during the dictator or generosity games. The participants played against remote players contacted prior to the study. Both players 1 and 2 were compensated in two randomly selected trials, so that each decision affected the welfare of both the decision-maker and another player.

The participants played the games alone in a closed room, unobserved and unrecorded. They were probed to ensure that they understood the tasks correctly. At the end of the game, each participant was informed of the identification number of the remote player who, as player 2, would receive monetary compensation in randomly selected trials. Next, all the participants completed a survey of their experiences during the games and cTBS, during which they were asked whether they believed that the remote players (player 2) were real people and had received their monetary compensation. Participants who expressed disbelief at any step of the study were expected to be excluded from the study.

None of the participants received monetary compensation for completion of the survey. In addition, both before and during the briefing, we made it clear to the participants that the study protocol involved no deception. At the end of the study, the subjects were paid based on the cumulative payoff from two randomly selected trials. None of the participants expressed disbelief in any steps of the study.

Each session consisted of 336 trials and lasted approximately 35 min. The trials in the generosity and dictator games were randomized, with the participants in each trial randomly taking part in the dictator game or generosity game. The participants’ payoffs consisted of a participation fee (approximately 4 U.S. dollars) and a monetary outcome in two randomly selected trials in the games. The participants collected 6 MUs (approximately 2 U.S. dollars) in the generosity game and 0–17 MUs (approximately 0–6 U.S. dollars) from each session of the dictator game. Thus, at the end of the study, the subjects were paid based on the cumulative payoff from two randomly selected trials. We ensured that the payoff matched the standard payoff in the original study of the generosity game^26^. In 2019, the Big Mac index estimated the price of a Big Mac in the U.S. at $5.74 and in Russia at $2 (130 rubles). The participants received 250 RUB (approximately equal to the cost of two Big Macs) as a participation fee and, in total, were able to collect a sum equal to 960 RUB (approximately equal to the cost of four Big Macs). The experiment was programmed using E-Prime 2.0 software.

The results of the dictator and generosity games and the split trials in the dictator game were compared in a post-hoc analysis. The trials in the dictator game were split into two trial types: trials with small endowments of between 7 and 11 MUs (similar to the pie sizes in the FP1 trials) and trials with large endowments of between 13 and 17 MUs (similar to the pie sizes in the FP2 trials).

### TMS

Previous research demonstrated that cortical excitability is suppressed for 60 min after 40 s of cTBS^53^. Thus, a short protocol of offline TMS, specifically cTBS^53^, was applied 5–7 min before the dictator and generosity games. cTBS was applied to the rDLPFC (main rDLPFC-TMS condition) or the rMT/V5 area (control rMT/V5-cTBS condition).

All the participants underwent an MRI scan to allow the use of navigated cTBS. The participants were seated in a comfortable armchair. TMS stimulation was delivered using a figure-of-eight coil, which was oriented posteriorly at a 45° angle from the midline and powered by a MagPro X100 stimulator (MagVenture, Inc.). The stimulation sites in the rDLPFC and MV/V5 were selected based on those in a previous TMS study^14^, as follows: for the rDLPFC, center of mass at MNI coordinates (x, y, z) = [24, 38, 40]; for the rMT/V5, (x, y, z) = [48, − 74, 0]. The control site (MRI coordinates) was selected based on the study by Christov-Moore et al.^14^, which demonstrated no effect of cTBS stimulation of the cortical rMT/V5 area on monetary transfers during the dictator game.

Motor evoked potentials were recorded using self-adhesive Ag–AgCl gel electrodes. In accordance with previous studies^8,14,54^, the MT was measured in the first dorsal interosseous of the left hand in the rDLPFC and rMT/V5 groups. The rMT was measured as described previously^55^. The cTBS protocol of TMS consisted of a train of pulse triplets delivered at 50 Hz, with the pulses repeated at 5 Hz, for a total of 600 pulses over 40 s, with the intensity set at 80% MT using a figure-of-eight coil, as described previously^14,54^. As current safety guidelines recommend not exceeding an intensity of 100% rMT^56^, in our study, the intensity was set at 80% rMT. A previous study that specifically addressed the safety of a similar cTBS protocol (with 80% rMT intensity) demonstrated no serious adverse effects^57^. We observed no side effects of cTBS in the current study. The participants evaluated seven types of sensations (itching, pain, burning, heating, pinching, a metallic taste in the mouth, and fatigue) during cTBS using a 5-point Likert scale, where 0 denoted “I did not feel this at all,” 1 denoted “I slightly felt this sensation,” 2 denoted “I felt this sensation”, 3 denoted “I felt this sensation moderately,” and 4 denoted “I felt this sensation very strongly.” No subject reported a strong sensation (level 4) during the cTBS stimulations.

We also checked differences in sensation levels between the stimulation groups using nonparametric statistics. The Mann − Whitney *U* test revealed no significant difference between cTBS of the rDLPFC and cTBS of the rMT/V5 for any type of sensation: itching, *U* = 256, *Z* =  − 0.270, *p* = 0.787; pain, *U* = 250, *Z* =  − 0.404, *p* = 0.686; burning, *U* = 243, *Z* =  − 0.967, *p* = 0.334; heating, *U* = 260,5, *Z* =  − 0.133, *p* = 0.894; pinching, *U* = 218,5, *Z* =  − 1.732, *p* = 0.083; a metallic taste in the mouth, *U* = 253, *Z* =  − 1.00, *p* = 0.317; and fatigue, *U* = 263.5, *Z* =  − 0.024, *p* = 0.981. We noticed a slightly but insignificantly higher pinching sensation after cTBS of the rMT/V5 than after cTBS of the rDLPFC (see details in supplementary Table 3).

### Statistical analysis

The normality of the data distribution was tested using a Kolmogorov–Smirnov test. The test showed that the offers in both stimulation groups were not normally distributed: rDLPFC group, p = 2.9 × 10^−223^; MT/V5 group, p = 1.2 × 10^−214^. Therefore, ordinal regression using the PLUM procedure was employed for parameter estimation (see^58,59^ as examples of the same approach), and a nonparametric Mann–Whitney *U*-test was used for a post-hoc analysis. First, the main factors *Stimulation* (cTBS of the rDLPFC vs. cTBS of the MT/V5) and *Game* (generosity game vs. dictator game) and the interaction of *Stimulation* × *Game* were analyses using ordinal regression (Model 1). Next, an additional post-hoc analysis was performed for the different trial types (FP2 trials vs. FP1 trials). The significance level was set at α ≤ 0.05. The data were analyzed using IBM SPSS 22.

The trials in the two games differed in terms of possible equity motivations. In the FP1 trials in the generosity game, preferences for generosity and equity were aligned. In contrast, in the FP2 trials, equity seeking and generosity favored different optimal allocations to player 2. To analyze the effect of *Stimulation* (cTBS of the rDLPFC vs. cTBS of the MT/V5)(rDLPFC vs. MT/V5) in the specific trial types (FP1 and FP2 trials) in the dictator and generosity games, the data were transformed, using a new ordinal regression model in which each level of the dependent variable (allocations to player 2) represented the trial type and game type (FP1 trials in the dictator game, FP1 trials in the generosity game, FP1 trials in the dictator game, and FP2 trials in the generosity game). We then fitted the model (Model 2) to the data with the factor *Stimulation (*rDLPFC vs. MT/V5). In the second analysis, two factors were tested in the two games: *Stimulation* (rDLPFC vs. MT/V5) and *Trial type* (FP1 trials in the dictator game, FP1 trials in the generosity game, FP1 trials in the dictator game, and FP2 trials in the generosity game). To correct for the multiple comparisons, we used Bonferroni correction (0.05/4 = 0.00125).

## Supplementary Information

Supplementary Tables.

## Data Availability

The dataset and script are available at Harvard Dataverse (https://dataverse.harvard.edu/dataverse/tmsstudy2020). Supplementary materials are available on the Open Science Framework website (https://osf.io/twd7f/).
