# Supplementary material for: Role of the prefrontal cortex in prosocial and self-maximization motivations: an rTMS study
Source: Sci Rep. 2021 Nov 16;11:22334. doi: 10.1038/s41598-021-01588-6 (PMC8595880; doi:10.1038/s41598-021-01588-6)
Supplement: Supplementary file 1 — Supplementary Tables. [file 41598_2021_1588_MOESM1_ESM.docx]

**Supplementary Table S1.** Descriptive statistics (for Model 1), showing the allocations to the recipients in the dictator game and the offered pie size in the generosity game.

| **Game** | **Mean** | **SEM** | **CI high** | **CI low** |
| --- | --- | --- | --- | --- |
| *Dictator Game/rDLPFC Stimulation* | 4.9896 | 0.036 | 5.0590 | 4.9203 |
| *Dictator Game/rMT/V5 Stimulation* | 4.6306 | 0.033 | 4.6953 | 4.5659 |
| *Generosity Game/rDLPFC Stimulation* | 7.3128 | 0.078 | 7.4645 | 7.1611 |
| *Generosity Game/rMT/V5 Stimulation* | 7.5543 | 0.076 | 7.7023 | 7.4064 |

**Supplementary Table S2.** Descriptive statistics (for Model 2), showing the allocations to the recipients in the dictator game and the offered pie size in the generosity game.

| **Trial Type** | **Mean** | **SEM** | **CI high** | **CI low** |
| --- | --- | --- | --- | --- |
| *FP1 in the Dictator Game rDLPFC Stimulation* | 3.8344 | 0.041 | 3.9140 | 3.7548 |
| *FP1 in the Generosity Game/rDLPFC Stimulation* | 4.3859 | 0.053 | 4.4897 | 4.2820 |
| *FP2 in the Dictator Game rDLPFC Stimulation* | 5.9523 | 0.050 | 6.0493 | 5.8553 |
| *FP2 in the Generosity Game/rDLPFC Stimulation* | 9.3134 | 0.080 | 9.4688 | 9.1580 |
| *FP1 in the Dictator Game/rMT/V5 Stimulation* | 3.4736 | 0.034 | 3.5390 | 3.4080 |
| *FP1 in the Generosity Game/rMT/V5 Stimulation* | 4.3750 | 0.046 | 4.4644 | 4.2856 |
| *FP2 in the Dictator Game/rMT/V5 Stimulation* | 5.5948 | 0.048 | 5.6884 | 5.5012 |
| *FP2 in the Generosity Game/rMT/V5 Stimulation* | 9.6159 | 0.070 | 9.7524 | 9.4795 |

**Supplementary Table 3.** Individuals’ discomfort ratings in the stimulations.

|  | **Sensation type** | | | | | | |  |
| --- | --- | --- | --- | --- | --- | --- | --- | --- |
| **Subject No.** | **Itching** | **Pain** | **Burning** | **Heating** | **Pinching** | **Metallic taste in mouth** | **Fatigue** | **Stimulation** |
| 1 | 0 | 1 | 0 | 0 | 0 | 0 | 2 | I |
| 2 | 1 | 1 | 0 | 0 | 1 | 0 | 1 | I |
| 3 | 0 | 0 | 0 | 0 | 0 | 0 | 0 | I |
| 4 | 0 | 0 | 0 | 0 | 0 | 0 | 0 | II |
| 5 | 0 | 1 | 0 | 0 | 0 | 0 | 1 | I |
| 6 | 0 | 1 | 0 | 0 | 1 | 0 | 0 | II |
| 7 | 0 | 1 | 0 | 1 | 0 | 0 | 2 | II |
| 8 | 0 | 0 | 0 | 0 | 0 | 0 | 3 | II |
| 9 | 1 | 1 | 0 | 0 | 1 | 0 | 2 | II |
| 10 | 0 | 0 | 0 | 0 | 0 | 0 | 1 | II |
| 11 | 0 | 1 | 0 | 2 | 0 | 0 | 1 | I |
| 12 | 0 | 0 | 0 | 0 | 0 | 0 | 0 | I |
| 13 | 3 | 0 | 2 | 1 | 0 | 0 | 2 | I |
| 14 | 0 | 0 | 0 | 0 | 0 | 0 | 2 | I |
| 15 | 0 | 0 | 0 | 1 | 0 | 0 | 3 | II |
| 16 | 0 | 0 | 0 | 0 | 0 | 0 | 1 | I |
| 17 | 1 | 0 | 0 | 0 | 1 | 0 | 0 | II |
| 18 | 0 | 0 | 1 | 0 | 0 | 0 | 0 | II |
| 19 | 0 | 0 | 0 | 0 | 0 | 0 | 0 | I |
| 20 | 0 | 0 | 0 | 0 | 0 | 0 | 0 | I |
| 21 | 0 | 1 | 0 | 0 | 0 | 0 | 1 | I |
| 22 | 2 | 3 | 0 | 0 | 0 | 0 | 0 | II |
| 23 | 0 | 0 | 0 | 0 | 0 | 0 | 0 | I |
| 24 | 0 | 0 | 0 | 0 | 0 | 0 | 0 | II |
| 25 | 0 | 0 | 0 | 0 | 1 | 0 | 1 | II |
| 26 | 0 | 0 | 0 | 0 | 0 | 0 | 0 | I |
| 27 | 0 | 2 | 0 | 0 | 0 | 0 | 2 | II |
| 28 | 0 | 0 | 0 | 0 | 0 | 0 | 2 | I |
| 29 | 0 | 0 | 0 | 0 | 0 | 0 | 0 | II |
| 30 | 0 | 0 | 0 | 0 | 0 | 0 | 0 | I |
| 31 | 0 | 0 | 0 | 0 | 0 | 0 | 0 | II |
| 32 | 0 | 1 | 1 | 0 | 0 | 0 | 2 | II |
| 33 | 0 | 0 | 0 | 0 | 0 | 0 | 0 | I |
| 34 | 1 | 0 | 0 | 0 | 0 | 0 | 1 | I |
| 35 | 1 | 0 | 0 | 0 | 0 | 0 | 0 | II |
| 36 | 0 | 0 | 0 | 0 | 0 | 0 | 0 | I |
| 37 | 0 | 0 | 0 | 0 | 0 | 0 | 0 | I |
| 38 | 0 | 0 | 0 | 1 | 0 | 0 | 0 | II |
| 39 | 2 | 1 | 1 | 0 | 0 | 0 | 0 | II |
| 40 | 0 | 0 | 0 | 1 | 0 | 0 | 0 | I |
| 41 | 0 | 2 | 0 | 0 | 0 | 1 | 2 | I |
| 42 | 0 | 0 | 0 | 0 | 0 | 0 | 2 | II |
| 43 | 3 | 0 | 0 | 2 | 0 | 0 | 1 | I |
| 44 | 0 | 0 | 0 | 0 | 0 | 0 | 1 | II |
| 45 | 0 | 0 | 0 | 1 | 1 | 0 | 0 | II |
| 46 | 0 | 0 | 0 | 0 | 0 | 0 | 0 | II |

Note: I – cTBS of the rDLPFC, II – cTBS of the rMT/V5.
